# Supplementary material for: Kinematic signatures in reaching movements during spaceflight provide evidence that humans underestimate body mass in microgravity
Source: eLife. 2026 Jul 9;14:RP107472. doi: 10.7554/eLife.107472 (PMC13349383; doi:10.7554/eLife.107472)
Supplement: Supplementary file 1. [file elife-107472-supp1.docx]

**Supplementary File 1.** The schedule of experiment sessions.

| **Taikonauts** | **Pre-flight** | | **In-flight** | | | **Post-flight** | |
| --- | --- | --- | --- | --- | --- | --- | --- |
| **S1** | -43 | -24 | 39 | 74 | -- | 14 | 66 |
| **S2** | -43 | -24 | 39 | -- | -- | 14 | -- |
| **S3** | -43 | -24 | 39 | 74 | -- | 14 | 66 |
| **S4** | -157 | -60 | 32 | 96 | 131 | 18 | 73 |
| **S5** | -165 | -60 | 32 | 96 | 131 | 18 | 73 |
| **S6** | -165 | -60 | 32 | 96 | 131 | 18 | 73 |
| **S7** | -31 | -12 | 20 | 109 | -- | 15 | 64 |
| **S8** | -31 | -12 | 20 | 109 | -- | 15 | 64 |
| **S9** | -31 | -12 | 20 | 109 | -- | 15 | 64 |
| **S10** | -91 | -48 | 28 | 76 | 125 | 16 | 93 |
| **S11** | -91 | -50 | 28 | 77 | 125 | 16 | 93 |
| **S12** | -91 | -52 | 28 | 76 | 125 | 16 | 93 |
| *Numbers in the table represent days before launch (pre-flight), days after launch (in-flight), and days after landing (post-flight).* | | | | | | | |
