# Supplementary material for: Kinematic signatures in reaching movements during spaceflight provide evidence that humans underestimate body mass in microgravity
Source: eLife. 2026 Jul 9;14:RP107472. doi: 10.7554/eLife.107472 (PMC13349383; doi:10.7554/eLife.107472)
Supplement: Supplementary file 2. [file elife-107472-supp2.docx]

**Supplementary File 2.** Group-level summary of the taikonauts' performance measures.

|  |  | pre | | | in | | | post | | |
| --- | --- | --- | --- | --- | --- | --- | --- | --- | --- | --- |
|  |  | 45^o^ | 90^o^ | 135^o^ | 45^o^ | 90^o^ | 135^o^ | 45^o^ | 90^o^ | 135^o^ |
| Reaction time with beep (ms) | mean | 267.353 | 248.361 | 247.929 | 262.117 | 242.279 | 241.056 | 267.830 | 245.480 | 238.843 |
|  | SE | 7.029 | 5.260 | 6.237 | 5.189 | 3.945 | 5.533 | 6.376 | 4.623 | 5.259 |
| Reaction time no beep (ms) | mean | 301.867 | 284.942 | 289.441 | 293.233 | 272.880 | 275.193 | 299.929 | 278.738 | 277.709 |
|  | SE | 6.518 | 5.481 | 6.747 | 4.559 | 4.748 | 5.259 | 5.576 | 4.873 | 4.629 |
| Movement duration (ms) | mean | 317.063 | 357.025 | 372.306 | 333.263 | 382.715 | 389.825 | 324.449 | 370.235 | 378.759 |
|  | SE | 10.964 | 8.239 | 6.332 | 8.209 | 7.511 | 6.943 | 7.005 | 6.114 | 5.609 |
| Peak acceleration (cm/s^2^) | mean | 1189.239 | 782.691 | 699.334 | 987.547 | 648.076 | 610.414 | 1067.384 | 699.108 | 670.281 |
|  | SE | 84.637 | 48.568 | 28.185 | 51.495 | 23.871 | 27.278 | 36.163 | 17.358 | 18.853 |
| Peak speed (cm/s) | mean | 90.528 | 76.508 | 68.820 | 82.910 | 69.198 | 63.574 | 86.143 | 74.280 | 67.412 |
|  | SE | 3.458 | 2.255 | 1.806 | 2.672 | 1.172 | 1.484 | 1.469 | 0.758 | 0.631 |
| Peak acc time (ms) | mean | 87.130 | 105.715 | 102.750 | 90.375 | 98.936 | 97.918 | 89.459 | 113.953 | 106.350 |
|  | SE | 3.348 | 4.479 | 2.388 | 1.668 | 2.453 | 2.089 | 2.646 | 5.938 | 3.205 |
| Peak speed time (ms) | mean | 143.789 | 166.768 | 183.458 | 150.217 | 167.623 | 183.281 | 148.776 | 171.886 | 180.029 |
|  | SE | 4.880 | 3.947 | 4.345 | 3.462 | 2.475 | 4.331 | 2.655 | 3.136 | 4.406 |
| Rel time to peak acc (%) | mean | 28.500 | 30.750 | 28.500 | 28.250 | 26.750 | 26.167 | 28.125 | 31.250 | 28.875 |
|  | SE | 0.413 | 0.854 | 0.609 | 0.664 | 0.698 | 0.474 | 0.453 | 1.213 | 0.640 |
| Rel time to peak speed (%) | mean | 46.250 | 47.583 | 50.458 | 46.208 | 44.875 | 47.917 | 46.750 | 47.000 | 48.083 |
|  | SE | 0.579 | 0.583 | 1.208 | 1.072 | 0.723 | 1.009 | 0.629 | 0.685 | 1.264 |
| Submovements (%) | mean | 0.692 | 0.717 | 0.592 | 0.764 | 0.855 | 0.763 | 0.670 | 0.652 | 0.632 |
|  | SE | 0.036 | 0.043 | 0.053 | 0.031 | 0.031 | 0.048 | 0.037 | 0.057 | 0.061 |
| Inter-peak-interval (ms) | mean | 57.600 | 67.856 | 60.918 | 60.471 | 85.788 | 77.527 | 56.385 | 63.976 | 61.525 |
|  | SE | 2.820 | 3.681 | 5.550 | 3.486 | 2.865 | 4.528 | 3.035 | 4.537 | 5.536 |
| Peak speed of 1st sub (cm/s) | mean | 75.046 | 60.964 | 54.520 | 66.718 | 54.049 | 47.379 | 71.538 | 59.791 | 53.892 |
|  | SE | 3.054 | 1.778 | 2.818 | 2.917 | 0.980 | 1.730 | 1.964 | 1.448 | 1.911 |
| Peak speed time of 1st sub (ms) | mean | 141.995 | 162.466 | 177.990 | 147.279 | 160.148 | 167.381 | 147.047 | 170.985 | 178.798 |
|  | SE | 5.074 | 4.239 | 5.091 | 2.572 | 1.727 | 5.597 | 4.213 | 4.870 | 5.270 |

*Values are group means and standard errors (SE) across the taikonauts, reported for each flight phase (pre-flight, in-flight, post-flight) and target direction (45°, 90°, 135°). These values underlie Figure 2-5 and statistical analyses presented in the main text.*

|  |  | pre-in | | | post-in | | |
| --- | --- | --- | --- | --- | --- | --- | --- |
| Δ peak speed (cm/s) | mean | 7.618 | 7.310 | 5.246 | 3.232 | 5.082 | 3.838 |
|  | SE | 1.961 | 1.723 | 1.447 | 2.220 | 1.291 | 1.294 |
| Δ peak speed time (ms) | mean | -5.284 | 2.317 | 10.608 | -0.232 | 10.836 | 11.416 |
|  | SE | 3.516 | 3.651 | 5.423 | 2.204 | 4.360 | 2.748 |
| Δ peak acc (cm/s2) | mean | 201.692 | 134.615 | 88.921 | 79.838 | 51.032 | 59.868 |
|  | SE | 58.848 | 41.791 | 23.751 | 39.882 | 24.523 | 23.915 |
| Δ peak acc time (ms) | mean | -3.245 | 6.779 | 4.832 | -0.916 | 15.017 | 8.431 |
|  | SE | 2.586 | 3.885 | 2.401 | 1.683 | 4.575 | 2.532 |

*Values are group means and standard errors (SE) of the change in each measure relative to in-flight, reported for each comparison (pre-in and post- -in flight) and target direction (45°, 90°, 135°). These values underlie Figure 6 and statistical analyses presented in the main text.*
