# Supplementary material for: Kinematic signatures in reaching movements during spaceflight provide evidence that humans underestimate body mass in microgravity
Source: eLife. 2026 Jul 9;14:RP107472. doi: 10.7554/eLife.107472 (PMC13349383; doi:10.7554/eLife.107472)
Supplement: Peer review history

# Editor report: eLife Assessment

**DOI:** 10.7554/eLife.107472.4.sa0

**Authors:** Alaa A Ahmed

**Affiliations:** University of Colorado Boulder United States

**Keywords:** Important, Solid

The authors present a **solid** study in the unique conditions of weightlessness providing evidence that movements carried out in 0g are underactuated. They further provide a thorough discussion based on computational modelling to address the question as to whether the CNS underestimates mass when programming movements in weightlessness. In all cases, the persistence of the observed effects in weightlessness has **important** implications for theories of motor adaptation.

# Referee report: Reviewer #1 (Public review)

**DOI:** 10.7554/eLife.107472.4.sa1

The authors have conducted substantial additional analyses to address the reviewers' comments. However, several key points still require attention. I was unable to see the correspondence between the model predictions and the data in the added quantitative analysis. In the rebuttal letter, the delta peak speed time displays values in the range of [20, 30] ms, whereas the data were negative for the 45{degree sign} direction. Should the reader directly compare panel B of Figure 6 with Figure 1E? The correspondence between the model and the data should be made more apparent in Figure 6. Furthermore, the rebuttal states that a quantitative prediction was not expected, yet it subsequently argues that there was a quantitative match. Overall, this response remains unclear.

A follow-up question concerns the argument about strategic slowing. The authors argue that this explanation can be rejected because the timing of peak speed should be delayed, contrary to the data. However, there appears to be a sign difference between the model and the data for the 45{degree sign} direction, which means that it was delayed in this case. Did I understand correctly? In that regard, I believe that the hypothesis of strategic slowing cannot yet be firmly rejected and the discussion should more clearly indicate that this argument is based on some, but not all, directions. I agree with the authors on the importance of the mass underestimation hypothesis, and I am not particularly committed to the strategic slowing explanation, but I do not see a strong argument against it. If the conclusion relies on the sign of the delta peak speed, then the authors' claims are not valid across all directions, and greater caution in the interpretation and discussion is warranted. Regarding the peak acceleration time, I would be hesitant to draw firm conclusions based on differences smaller than 10 ms (Figures R3 and 6D).

The authors state in the rebuttal that the two hypotheses are competing. This is not accurate, as they are not mutually exclusive and could even vary as a function of movement direction. The abstract also claims that the data "refutes" strategic slowing, which I believe is too strong. The main issue is that, based on the authors' revised manuscript, the lack of quantitative agreement between the model and the data for the mass underestimation hypothesis is considered acceptable because a precise quantitative match is not expected, and the predictions overall agree for some (though not all) directions and phases (excluding post-in). That is reasonable, but by the same logic, the small differences between the model prediction and the strategic slowing hypothesis should not be taken as firm evidence against it, as the authors seem to suggest. In practice, I recommend a more transparent and cautious interpretation to avoid giving readers the false impression that the evidence is decisive. The mass underestimation hypothesis is clearly supported, but the remaining aspects are less clear, and several features of the data remain unexplained.

Comments on revised version.

The authors have reworked the sections of the text where the narrative was too strong or binary wrt alternative interpretations. The result is well balanced. No further recommendation.

# Referee report: Reviewer #3 (Public review)

**DOI:** 10.7554/eLife.107472.4.sa2

Summary:

The authors describe an interesting study of arm movements carried out in weightlessness after a prolonged exposure to the so-called microgravity conditions of orbital spaceflight. Subjects performed radial point-to-point motions of the fingertip on a touch pad. The authors note a reduction in movement speed in weightlessness, which they hypothesize could be due to either an overall strategy of lowering movement speed to better accommodate the instability of the body in weightlessness or an underestimation of body mass. They conclude for the latter, mainly based on two effects. One, slowing in weightlessness is greater for movement directions with higher effective mass at the end effector of the arm. Two, they present evidence for increased number of corrective sub movements in weightlessness. They contend that this provides conclusive evidence to accept the hypothesis of an underestimation of body mass.

Strengths:

In my opinion, the study provides a valuable contribution, the theoretical aspects are well presented through simulations, the statistical analyses are meticulous, the applicable literature is comprehensively considered and cited and the manuscript is well written.

Weaknesses:

I nevertheless am of the opinion that the interpretation of the observations leaves room for other possible explanations of the observed phenomenon, thus weakening the strength of the arguments.

I raised the following points in my original review, but I find that the authors have judiciously addressed these points through their various revisions.

I believe that the article constitutes a valuable contribution and that the results and conclusions are certainly worthy of consideration by the human motor control community.

(1) The authors model the movement control through equations that derive the input control variable in terms of the force acting on the hand and treating the arm as a second-order low pass filter (Eq. 13). Underestimation of the mass in the computation of a feedforward command would lead to a lower-than-expected displacement to that command. But it is not clear if and how the authors account for a potential modification of the time constants of the 2nd order system. The CNS does not effectuate movements with pure torque generators. Muscles have elastic properties that depend on their tonic excitation level, reflex feedback and other parameters. Indeed, Fisk et al.* showed variations of movement characteristics consistent with lower muscle tone, lower bandwidth and lower damping ratio in 0g compared to 1g. Could the variations in the response to the initial feedforward command be explained by a misrepresentation of the limbs damping and natural frequency, leading to greater uncertainty to the consequences of the initial command. This would still be an argument for un-adapted feedforward control of the movement, leading to the need for more corrective movements. But it would not necessarily reflect an underestimation of body mass.

*Fisk, J. O. H. N., Lackner, J. R., & DiZio, P. A. U. L. (1993). Gravitoinertial force level influences arm movement control. Journal of neurophysiology, 69(2), 504-511.

While the authors attempt to differentiate their study from previous studies where limb neuromechanical impedance was shown to be modified in weightlessness by emphasizing that in the current study the movements were rapid and the initial movement is "feedforward". But this incorrectly implies that the limb's mechanical response to the motor command is determined only by active feedback mechanisms. In fact:

(a) All commands to the muscle pass through the motor neurons. These neurons receive descending activations related not only to the volitional movement, but also to the dynamic state of the body and the influence of other sensory inputs, including the vestibular system. A decrease in descending influences from the vestibular organs will lower the background sensitivity to all other neural influences on the motor neuron. Thus, the motor neuron may be less sensitive to the other volitional and reflexive synaptic inputs that it may receive.

(b) Muscle tone plays a significant role in determining the force and the time course of the muscle contraction. In a weightless environment, where tonic muscle activity is likely to be reduced, there is the distinct possibility that muscles will react more slowly and with lower amplitude to an otherwise equivalent descending motor command, particularly in the initial moments before spinal reflexes come into play. These, and other neuronal mechanisms could lead to the "under-actuation" effect observed in the current study, without necessarily being reflective of an underestimation of mass per se.

(2) The subject's body in weightless is much more sensitive to reaction forces in interactions with the environment in the absence of the anchoring effect of gravity pushing the body into the floor and in the absence of anticipatory postural adjustments that typically accompany upper-limb motions in Earth gravity in order to maintain an upright posture. The authors dismiss this possibility because the taikonauts were asked to stabilize their bodies with the contralateral hand. But the authors present no evidence that this was sufficient to maintain the shoulder and trunk at a strictly constant position, as is supposed by the simplified biomechanical model used in their optimal control framework. Indeed, a small backward motion of the shoulder would result in a smaller acceleration of the fingertip and a smaller extent of the initial ballistic motion of the hand with respect to the measurement device (the tablet), consistent with the observations reported in the study. Note that stability of the base might explain why 45º movements were apparently less affected in weightlessness, according to many of the reported analyses, including those related to corrective movements (Fig. 5 B, C, F; Fig. 6D), than the other two directions. If the trunk is being stabilized by the left arm, the same reaction forces on the trunk due to the acceleration of the hand will result in less effective torque on the trunk, given that the reaction forces act with a much smaller moment arm with respect to the left shoulder (the hand movement axis passes approximately through the left shoulder for the 45º target) compared to either the forward or rightward motions of the hand.

(3) The above is exacerbated by potential changes in the frictional forces between the fingertip and the tablet. The movements were measured by having the subjects slide their finger on the surface of a touch screen. In weightlessness, the implications of this contact can be expected to be quite different than on the ground. While these forces may be low on Earth, the fact is that we do not know what forces the taikonauts used on orbit. In weightlessness, the taikonauts would need to actively press downward to maintain contact with the screen, while on Earth gravity will do the work. The tangential forces that resist movement due to friction might therefore be different in 0g. . Indeed, given the increased instability of the body and the increased uncertainty of movement direction of the hand, taikonauts may have been induced to apply greater forces against the tablet in order to maintain contact in weightlessness, which would in turn slow the motion of the finger on the table and increase the reaction forces acting on the trunk. This could be particularly relevant given that the effect of friction would interact with the limb in a direction-dependent fashion, given the anisotropy of the equivalent mass at the fingertip evoked by the authors.

I feel that the authors have done an admirable job of exploring the how to explain the modifications to movement kinematics that they observed on orbit within the constraints of the optimal control theory applied to a simplified model of the human motor system. While I fully appreciate the value of such models to provide insights into question of human sensorimotor behaviour, to draw firm conclusions on what humans are actually experiencing based only on manipulations of the computational model, without testing the model's implicit assumptions and without considering the actual neurophysiological and biomechanical mechanisms, can be misleading. One way to do this could be to examine these questions through extensions to the model used in the simulations (changing activation dynamics of the torque generators, allowing for potential motion backward motion of the shoulder and trunk, etc.). A better solution would be to emulate the physiological and biomechanical conditions on Earth (supporting the arm against gravity to reduce muscle tone, placing the subject on a moveable base that requires that the body be stabilized with the other hand) in order to distinguish the hypothesis of an underestimation of mass vs. other potential sources of under-actuation and other potential effects of weightlessness on the body.

In sum, my opinion is that the authors are relying too much on a theoretical model as a ground truth and thus overstate their conclusions. But to provide a convincing argument that humans truly underestimate mass in weightlessness, they should consider more judiciously the neurophysiology and biomechanics that fall outside the purview of the simplified model that they have chosen. If a more thorough assessment of this nature is not possible, then I would argue that a more measured conclusion of the paper should be (1) that the authors observed modifications to movement kinematics in weightlessness consistent with an under-actuation for the intended motion, (2) that a simplified model of human physiology and biomechanics that incorporates principles of optimal control suggest that the source of this under-actuation might be an underestimation of mass in the computation of an appropriate feedforward motor command, and (3) that other potential neurophysiological or biomechanical effects cannot be excluded due to limitations of the computational model.

# Author comment: Author response

**DOI:** 10.7554/eLife.107472.4.sa3

**Authors:** Zhaoran Zhang, Yu Tian, Chunhui Wang, Changhua Jiang, Bo Wang, Hongqiang Yu, Rui Zhao, Kunlin Wei

**Affiliations:** Shenzhen University Shenzhen China; China Astronaut Research and Training Center Beijing China; China Astronaut Research and Training Center Beijing China; China Astronaut Research and Training Center Beijing China; China Astronaut Research and Training Center Beijing China; China Astronaut Research and Training Center Beijing China; China Astronaut Research and Training Center Beijing China; Peking University Beijing China

The following is the authors’ response to the original reviews.

**General recommendations (from the Reviewing Editor):**

The reviewers discussed the revision at length, and all were appreciative of the revisions to the paper. Nonetheless, they agreed that the evidence against alternative hypotheses was not yet decisive, and it may not be possible to provide the evidence needed given the difficulty of acquiring this data. Thus they feel that a more nuanced interpretation of the data and tempering of the conclusions is necessary. These points are described in more detail in the reviewer-specific comments in the Public reviews.

We thank the editor and the reviewers for their constructive discussion. In this revision, we have adopted these recommendations: we have tempered our conclusions and removed binary framing, taking into consideration that other alternative explanations might exist. We have also expanded the Discussion to consider additional potential mechanisms and added corresponding limitations. We also changed the paper title to avoid strong inference; the new title is “Evidence that humans underestimate body mass in microgravity: kinematic signatures in reaching movements during spaceflight”.

**Public Reviews:**

**Reviewer #1 (Public review):**

The authors have conducted substantial additional analyses to address the reviewers' comments. However, several key points still require attention. I was unable to see the correspondence between the model predictions and the data in the added quantitative analysis. In the rebuttal letter, the delta peak speed time displays values in the range of [20, 30] ms, whereas the data were negative for the 45{degree sign} direction. Should the reader directly compare panel B of Figure 6 with Figure 1E? The correspondence between the model and the data should be made more apparent in Figure 6. Furthermore, the rebuttal states that a quantitative prediction was not expected, yet it subsequently argues that there was a quantitative match. Overall, this response remains unclear.

We thank the reviewer raising the question about Figure 6B. We would like to clarify that the phrase "quantitative match" in the summary of our previous rebuttal letter was a wording error; in fact, the subsequent detailed responses consistently and correctly described the comparison as qualitative. We apologize for the confusion this may have caused, and address this point below.

First, we have revised the manuscript to clarify this point. We have added the following statement: "We note that these correlations evaluate the directional trend rather than the absolute magnitude of the effects; a precise quantitative match is not expected given the simplifications of the two-joint arm model." in the main text.

Second, we have replaced Figure 6 with a revised version that presents model-predicted Δ values and experimentally observed Δ values side by side, allowing for a more intuitive visual comparison. As shown in the updated figure, the directional trends are broadly consistent amplitude changes and timing shifts are rank-ordered by movement direction in both model and data while the absolute magnitudes do not precisely match. We believe this layout makes the intended comparison more transparent.

As discussed in our previous response, as noted above, a precise quantitative match is not expected given our model's simplifications, and this level of qualitative comparison is consistent with established practice in similar modeling studies (e.g., Gaveau et al., 2016).

Regarding the negative Δ peak speed time at 45°: as shown in our statistical analyses (Figure 4A, Figure 5F), there was no significant timing change at 45°. The negative value reflects a small, non-significant mean difference. The key pattern that timing advance increases for directions associated with higher effective inertia holds for the 90° and 135° directions, which is the directional trend our analysis was designed to capture.

A follow-up question concerns the argument about strategic slowing. The authors argue that this explanation can be rejected because the timing of peak speed should be delayed, contrary to the data. However, there appears to be a sign difference between the model and the data for the 45{degree sign} direction, which means that it was delayed in this case. Did I understand correctly? In that regard, I believe that the hypothesis of strategic slowing cannot yet be firmly rejected and the discussion should more clearly indicate that this argument is based on some, but not all, directions.

I agree with the authors on the importance of the mass underestimation hypothesis, and I am not particularly committed to the strategic slowing explanation, but I do not see a strong argument against it. If the conclusion relies on the sign of the delta peak speed, then the authors' claims are not valid across all directions, and greater caution in the interpretation and discussion is warranted. Regarding the peak acceleration time, I would be hesitant to draw firm conclusions based on differences smaller than 10 ms (Figures R3 and 6D).

The authors state in the rebuttal that the two hypotheses are competing. This is not accurate, as they are not mutually exclusive and could even vary as a function of movement direction. The abstract also claims that the data "refutes" strategic slowing, which I believe is too strong. The main issue is that, based on the authors' revised manuscript, the lack of quantitative agreement between the model and the data for the mass underestimation hypothesis is considered acceptable because a precise quantitative match is not expected, and the predictions overall agree for some (though not all) directions and phases (excluding post-in). That is reasonable, but by the same logic, the small differences between the model prediction and the strategic slowing hypothesis should not be taken as firm evidence against it, as the authors seem to suggest. In practice, I recommend a more transparent and cautious interpretation to avoid giving readers the false impression that the evidence is decisive. The mass underestimation hypothesis is clearly supported, but the remaining aspects are less clear, and several features of the data remain unexplained.

We thank the reviewer for this critical assessment. We acknowledge that our previous framing was too binary, and we agree that strategic slowing and mass underestimation are not mutually exclusive. We would like to clarify our view: we did not find evidence supporting strategic slowing (e.g., slower reaction times, symmetric velocity/acceleration peaks), whereas we did find evidence supporting mass underestimation (asymmetric peaks, unchanged reaction times, more sub movements). This is not a case of rejecting one hypothesis to affirm the other; our data simply do not support one while providing positive evidence for the other. We do not rule out the possibility that both mechanisms could operate together, though we note that our data did not reveal evidence supporting strategic slowing in the current reaching task.

We also agree that the lack of significant timing changes at 45° limits the scope of our argument against strategic slowing in that direction. However, the null result at 45° likewise cannot serve as positive evidence for strategic slowing either. As discussed in our previous revision and in Discussion, this null effect may arise because 45° reaches are predominantly single-joint (evidenced by curvature patterns characteristic), making them less suitable for modeling with a simplified two-link arm model than the 90° and 135° directions.

In line with these considerations, we have made the following revisions to the manuscript:

(1) We have removed binary framing throughout, replacing claims of mutual exclusivity or outright rejection of strategic slowing with more measured language. For example, "refutes" in the abstract has been changed to "These findings provide support for the body mass underestimation hypothesis while being inconsistent with the strategic slowing hypothesis." The two hypotheses are no longer presented as mutually exclusive, and strategic slowing is now characterized as insufficient to fully explain the direction-dependent pattern, rather than ruled out entirely.

(2) We have revised the conclusion. The concluding paragraph no longer presents an either-or outcome. We describe the direction-dependent under-actuation pattern, note that it strongly supports mass underestimation while not being readily explained by a uniform strategic adjustment, and acknowledge that other factors may also contribute. A new limitation paragraph discusses the simplified nature of our model and acknowledges that other neurophysiological and biomechanical factors cannot be excluded.

**Reviewer #2 (Public review):**

This study explores the underlying causes of the generalized movement slowness observed in astronauts in weightlessness compared to their performance on Earth. The authors argue that this movement slowness stems from an underestimation of mass rather than a deliberate reduction in speed for enhanced stability and safety.

Overall, this is a fascinating and well-written work. The kinematic analysis is thorough and comprehensive. The design of the study is solid, the collected dataset is rare, and the model adds confidence to the proposed conclusions.

Compared to the previous version, the authors have thoroughly addressed my concerns. The model is now clear and well-articulated, and alternative hypotheses have been ruled out convincingly. The paper is improved and suitable for publication in my opinion, making a significant contribution to the field.

Strengths:

Comprehensive analysis of a unique data set of reaching movement in microgravity

Use of a sensible and well-thought experimental approach

State-of-the-art analyses of main kinematic parameter

Computational model simulations of arm reaching to test alternative hypotheses and support the mass underestimation one

This work has no major weakness as it stands, and the discussion provides a fair evaluation of the findings and conclusions.

We thank the reviewer for the supportive feedback, and we are grateful for the earlier comments that helped us improve the manuscript.

**Reviewer #3 (Public review):**

Summary:

The authors describe an interesting study of arm movements carried out in weightlessness after a prolonged exposure to the so-called microgravity conditions of orbital spaceflight. Subjects performed radial point-to-point motions of the fingertip on a touch pad. The authors note a reduction in movement speed in weightlessness, which they hypothesize could be due to either an overall strategy of lowering movement speed to better accommodate the instability of the body in weightlessness or an underestimation of body mass. They conclude for the latter, mainly based on two effects. One, slowing in weightlessness is greater for movement directions with higher effective mass at the end effector of the arm. Two, they present evidence for increased number of corrective submovements in weightlessness. They contend that this provides conclusive evidence to accept the hypothesis of an underestimation of body mass.

Strengths:

In my opinion, the study provides a valuable contribution, the theoretical aspects are well presented through simulations, the statistical analyses are meticulous, the applicable literature is comprehensively considered and cited and the manuscript is well written.

Weaknesses:

I nevertheless am of the opinion that the interpretation of the observations leaves room for other possible explanations of the observed phenomenon, thus weakening the strength of the arguments.

To strengthen the conclusions, I feel that the following points would need to be addressed:

We thank the reviewer for the insightful critique and constructive suggestions. Following the reviewer's advice, we have re-framed our Introduction and Discussion to present mass underestimation as a plausible mechanism identified by our simplified model, while explicitly acknowledging other potential factors. Below we address each point in detail.

(1) The authors model the movement control through equations that derive the input control variable in terms of the force acting on the hand and treating the arm as a second-order low pass filter (Eq. 13). Underestimation of the mass in the computation of a feedforward command would lead to a lower-than-expected displacement to that command. But it is not clear if and how the authors account for a potential modification of the time constants of the 2nd order system. The CNS does not effectuate movements with pure torque generators. Muscles have elastic properties that depend on their tonic excitation level, reflex feedback and other parameters. Indeed, Fisk et al.* showed variations of movement characteristics consistent with lower muscle tone, lower bandwidth and lower damping ratio in 0g compared to 1g. Could the variations in the response to the initial feedforward command be explained by a misrepresentation of the limbs damping and natural frequency, leading to greater uncertainty to the consequences of the initial command. This would still be an argument for un-adapted feedforward control of the movement, leading to the need for more corrective movements. But it would not necessarily reflect an underestimation of body mass.

*Fisk, J. O. H. N., Lackner, J. R., & DiZio, P. A. U. L. (1993). Gravitoinertial force level influences arm movement control. Journal of neurophysiology, 69(2), 504-511.

While the authors attempt to differentiate their study from previous studies where limb neuromechanical impedance was shown to be modified in weightlessness by emphasizing that in the current study the movements were rapid and the initial movement is "feedforward". But this incorrectly implies that the limb's mechanical response to the motor command is determined only by active feedback mechanisms. In fact:

(a) All commands to the muscle pass through the motor neurons. These neurons receive descending activations related not only to the volitional movement, but also to the dynamic state of the body and the influence of other sensory inputs, including the vestibular system. A decrease in descending influences from the vestibular organs will lower the background sensitivity to all other neural influences on the motor neuron. Thus, the motor neuron may be less sensitive to the other volitional and reflexive synaptic inputs that it may receive.

(b) Muscle tone plays a significant role in determining the force and the time course of the muscle contraction. In a weightless environment, where tonic muscle activity is likely to be reduced, there is the distinct possibility that muscles will react more slowly and with lower amplitude to an otherwise equivalent descending motor command, particularly in the initial moments before spinal reflexes come into play. These, and other neuronal mechanisms could lead to the "under-actuation" effect observed in the current study, without necessarily being reflective of an underestimation of mass per se.

The reviewer raises an important point that the observed underactuation may not necessarily reflect mass underestimation per se. It could also arise from changes in the time constants of the control system, tonic muscle activation levels, vestibular descending inputs, or altered spinal reflex gains. We agree that our simplified model does not capture these neuromuscular factors, and we have made several revisions to address this concern.

In the Discussion (paragraph 4), we have added a new substantive section discussing how reduced tonic muscle activity, diminished vestibular inputs to motor neurons, and altered muscle activation dynamics (Fisk et al., 1993) may contribute to the observed under-actuation independently of mass misestimation. We argue that while these factors likely affect motor output, they would be expected to produce a relatively uniform effect across movement directions, as tonic muscle activation and vestibular descending inputs are not specific to a particular reaching direction. In contrast, the direction-dependent pattern of our results with greater effects for directions involving higher effective mass is more naturally explained by a misrepresentation of inertial properties than by a uniform change in neuromuscular excitability. Nevertheless, we explicitly acknowledge that these mechanisms may act in concert with mass underestimation, and that our current data cannot fully disentangle them.

Additionally, the paragraph discussing proprioceptive mechanisms (paragraph 6 of Discussion) now opens with the conditional framing "If mass underestimation contributes to the observed underactuation," and closes by noting that the same proprioceptive degradation could affect motor output through other pathways such as reducing tonic muscle activation or altering spinal reflex gains independent of any explicit misrepresentation of body mass.

We have also added a new limitation (the fourth in the Limitations section) explicitly acknowledging that our model treats muscles as ideal torque generators and does not capture potential changes in muscle activation dynamics, damping, or reflex gains that may occur in microgravity. Future studies combining detailed musculoskeletal modeling with direct measurements of muscle activation, joint impedance, and trunk kinematics would be needed to distinguish between mass underestimation and other sources of underactuation.

That said, the assumption of relatively preserved muscle properties is partly supported by the available evidence. A systematic review of simulated microgravity studies found that upper limb maximal voluntary contraction remained mostly unchanged for up to 45 days of unloading, and that upper limb muscles declined substantially more slowly than lower limb and trunk muscles (Winnard et al., 2019). A more recent review similarly reported that upper limb muscle outcomes are less affected by microgravity exposure (Bosutti et al., 2025). This is also consistent with our own unpublished observations in Chinese astronauts, which did not indicate an obvious decline in upper limb force output. While these findings do not rule out subtler changes in muscle tone or activation dynamics, they suggest that gross alterations in upper limb neuromuscular capacity are unlikely to be the primary driver of the underactuation we observed.

Refs.

Winnard, A., Scott, J., Waters, N., Vance, M., & Caplan, N. (2019). Effect of time on human muscle outcomes during simulated microgravity exposure without countermeasures—systematic review. Frontiers in physiology, 10, 1046.

Bosutti, A., Ganse, B., Maffiuletti, N. A., Wüst, R. C., Strijkers, G. J., Sanderson, A., & Degens, H. (2025). Microgravity‐induced changes in skeletal muscle and possible countermeasures: What we can learn from bed rest and human space studies. Experimental Physiology.

(2) The subject's body in weightless is much more sensitive to reaction forces in interactions with the environment in the absence of the anchoring effect of gravity pushing the body into the floor and in the absence of anticipatory postural adjustments that typically accompany upper-limb motions in Earth gravity in order to maintain an upright posture. The authors dismiss this possibility because the taikonauts were asked to stabilize their bodies with the contralateral hand. But the authors present no evidence that this was sufficient to maintain the shoulder and trunk at a strictly constant position, as is supposed by the simplified biomechanical model used in their optimal control framework. Indeed, a small backward motion of the shoulder would result in a smaller acceleration of the fingertip and a smaller extent of the initial ballistic motion of the hand with respect to the measurement device (the tablet), consistent with the observations reported in the study. Note that stability of the base might explain why 45º movements were apparently less affected in weightlessness, according to many of the reported analyses, including those related to corrective movements (Fig. 5 B, C, F; Fig. 6D), than the other two directions. If the trunk is being stabilized by the left arm, the same reaction forces on the trunk due to the acceleration of the hand will result in less effective torque on the trunk, given that the reaction forces act with a much smaller moment arm with respect to the left shoulder (the hand movement axis passes approximately through the left shoulder for the 45º target) compared to either the forward or rightward motions of the hand.

The reviewer raises an important point about the potential influence of reaction forces on trunk and shoulder stability in microgravity. We have revised the relevant Discussion paragraph to address this concern more thoroughly.

We would like to clarify that, in addition to stabilizing the body with the left hand grasping a fixed bar, the taikonauts’ feet were also constrained with foot straps, providing multi-point stabilization. Furthermore, the reviewer's trunk displacement hypothesis predicts that the 45° direction should be systematically less affected across all kinematic measures. However, while 45° did not show significant changes in the timing of kinematics peaks, it did show significant changes in movement duration, peak acceleration, and peak speed comparable to the other directions. This dissociation is difficult to reconcile with a uniform trunk displacement artifact, but is consistent with a direction-dependent inertial effect.

We acknowledge that we did not directly measure trunk or shoulder kinematics, highlight that we did our best to provide multi-point stabilization in our setup, and we have added this as a limitation in the revised Discussion.

(3) The above is exacerbated by potential changes in the frictional forces between the fingertip and the tablet. The movements were measured by having the subjects slide their finger on the surface of a touch screen. In weightlessness, the implications of this contact can be expected to be quite different than on the ground. While these forces may be low on Earth, the fact is that we do not know what forces the taikonauts used on orbit. In weightlessness, the taikonauts would need to actively press downward to maintain contact with the screen, while on Earth gravity will do the work. The tangential forces that resist movement due to friction might therefore be different in 0g. . Indeed, given the increased instability of the body and the increased uncertainty of movement direction of the hand, taikonauts may have been induced to apply greater forces against the tablet in order to maintain contact in weightlessness, which would in turn slow the motion of the finger on the table and increase the reaction forces acting on the trunk. This could be particularly relevant given that the effect of friction would interact with the limb in a direction-dependent fashion, given the anisotropy of the equivalent mass at the fingertip evoked by the authors

We agree that in microgravity, taikonauts must actively press on the screen to maintain contact, potentially altering normal forces and thus friction compared to ground conditions. We have acknowledged this point in the revised Discussion. However, we note several reasons why friction is unlikely to be the dominant factor. First, the tablet uses a capacitive touchscreen, which registers touch through changes in electrical capacitance and does not require substantial normal force to maintain contact. Second, typical tangential friction forces during touchscreen interaction range from 0.1 to 0.5 N (Ayyildiz et al., 2018), which are small compared to the 10–15 N required to accelerate the arm during reaching. Third, touchscreen performance has been shown to be largely unaffected during long-duration spaceflight (Holden et al., 2022). Lastly but importantly, the friction hypothesis does not readily account for the direction-specific pattern of effects we observed. While we cannot exclude a contribution of altered friction, particularly in interaction with the direction-dependent effective mass, its magnitude makes it unlikely to account for the observed kinematic changes.

Ref:

Ayyildiz, M., Scaraggi, M., Sirin, O., Basdogan, C., & Persson, B. N. J. (2018). Contact mechanics between the human finger and a touchscreen under electroadhesion. Proceedings of the National Academy of Sciences of the United States of America, 115(50), 12668–12673.

Holden, K., Greene, M., Vincent Cross, E., Sandor, A., Thompson, S., Feiveson, A., & Munson, B. (2023). Effects of long-duration microgravity and gravitational transitions on fine motor skills. Human Factors, 65(6), 1046-1058.

I feel that the authors have done an admirable job of exploring the how to explain the modifications to movement kinematics that they observed on orbit within the constraints of the optimal control theory applied to a simplified model of the human motor system. While I fully appreciate the value of such models to provide insights into question of human sensorimotor behaviour, to draw firm conclusions on what humans are actually experiencing based only on manipulations of the computational model, without testing the model's implicit assumptions and without considering the actual neurophysiological and biomechanical mechanisms, can be misleading. One way to do this could be to examine these questions through extensions to the model used in the simulations (changing activation dynamics of the torque generators, allowing for potential motion backward motion of the shoulder and trunk, etc.). A better solution would be to emulate the physiological and biomechanical conditions on Earth (supporting the arm against gravity to reduce muscle tone, placing the subject on a moveable base that requires that the body be stabilized with the other hand) in order to distinguish the hypothesis of an underestimation of mass vs. other potential sources of under-actuation and other potential effects of weightlessness on the body.

In sum, my opinion is that the authors are relying too much on a theoretical model as a ground truth and thus overstate their conclusions. But to provide a convincing argument that humans truly underestimate mass in weightlessness, they should consider more judiciously the neurophysiology and biomechanics that fall outside the purview of the simplified model that they have chosen. If a more thorough assessment of this nature is not possible, then I would argue that a more measured conclusion of the paper should be (1) that the authors observed modifications to movement kinematics in weightlessness consistent with an under-actuation for the intended motion, (2) that a simplified model of human physiology and biomechanics that incorporates principles of optimal control suggest that the source of this under-actuation might be an underestimation of mass in the computation of an appropriate feedforward motor command, and (3) that other potential neurophysiological or biomechanical effects cannot be excluded due to limitations of the computational model.

We appreciate the reviewer's thoughtful assessment. We fully agree that a simplified computational model should not be treated as ground truth, and that the neurophysiology and biomechanics beyond the computational model must be carefully considered.

As detailed in our responses above, we have substantially revised the Discussion to address each of these concerns—including new discussions of neuromuscular factors, more balanced treatment of trunk stability and friction, conditional framing of the mass underestimation interpretation, and a new limitation on model simplifications. The conclusion has been restructured following the reviewer's recommended framework.

**Recommendations for the authors:**

**Reviewer #2 (Recommendations for the authors):**

If possible and allowed, the authors are strongly encouraged to consider sharing this unique dataset. Making the data publicly available alongside the paper could foster future studies and further accelerate research in this area.

We sincerely thank the reviewer for this suggestion. The ground control data and all analysis code will be made publicly available alongside the Version of Record.

However, unfortunately, the raw in-flight data from the taikonaut cohort cannot be made publicly available due to confidentiality regulations of China's manned space program; access for scientific research requires approval from the China Astronaut Research and Training Center and can be requested through the corresponding author.
